# The Enantiomeric Discrimination of 5-Hexyl-2-methyl-3,4-dihydro-2*H*-pyrrole by Sulfobutyl ether-β-cyclodextrin: A Case Study

**DOI:** 10.3390/molecules26092611

**Published:** 2021-04-29

**Authors:** Daniel F. Kawano, Bruna Z. Costa, Katherine L. Romero-Orejón, Hugo C. Loureiro, Dosil P. de Jesus, Anita J. Marsaioli

**Affiliations:** 1 Institute of Chemistry, University of Campinas, Campinas 13083-861, SP, Brazil; dkawano@unicamp.br (D.F.K.); bzucoloto@gmail.com (B.Z.C.); katherine.romero019@gmail.com (K.L.R.-O.); dosil@unicamp.br (D.P.d.J.); 2 Faculty of Pharmaceutical Sciences, University of Campinas, Campinas 13083-871, SP, Brazil

**Keywords:** chiral capillary electrophoresis, pyrroline, enantiodifferenciation, docking, sulfotbutyl ether-β-cyclodextrin

## Abstract

1-Pyrrolines are important intermediates of active natural products, such as the 2,5-dialkyl-1-pyrroline derivatives found in fire ant venoms. Here, 5-hexyl-2-methyl-3,4-dihydro-2*H*-pyrrole was synthesized by the enzymatic transamination/cyclization of 2,5-undecadione, and enantiodifferenciation was successfully achieved by capillary electrophoresis with sulfobutyl ether-β-cyclodextrin as the chiral selector. The rationale of the enantiomeric discrimination was based on the results of a docking simulation that revealed the higher affinity of (*S*)-5-hexyl-2-methyl-3,4-dihydro-2*H*-pyrrole for the sulfobutyl ether-β-cyclodextrin.

## 1. Introduction

**Figure 1:**
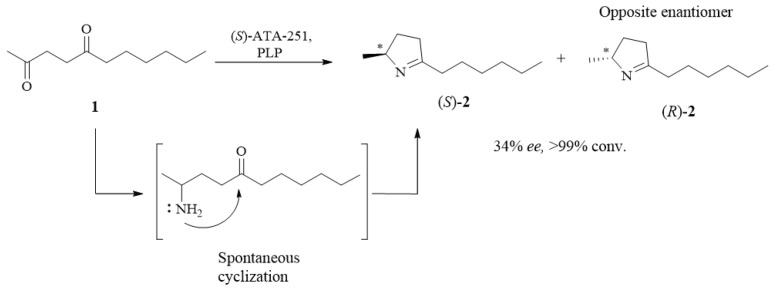
Reductive amination reaction of **1** by *S*-selective TA (ATA-251) or *R*-selective TA (ATA-117) to obtain enantiopure (*S*)-**2** and (*R*)-**2**, respectively. The products were obtained with excellent (>99%) yields and enantiomeric excess (*ee*).

2,5-Dialkyl-1-pyrrolines are the synthetic intermediates of several bioactive lipophilic alkaloids present in ants and frogs [1,2]. Usually, their asymmetric synthesis from 1,4-diketones **1** using enantioselective transaminases (TAs), which are dependent on pyridoxal-5${}^{\prime }$-phosphate (PLP), leads to enantiomerically enriched pyrrolines **2**, as shown in Figure 1. The predominant enantiomer absolute configuration is assumed to follow the transaminase preference. However, additional evidence should be obtained during the absolute configuration evaluation of the enantiomers to prevent future configuration problems.

The enantiomeric discrimination of 5-hexyl-2-methyl-3,4-dihydro-2*H*-pyrrole **2** was first tested with gas chromatography–mass spectrometry (GC–MS) equipped with a chiral capillary column of β-cyclodextrin, which unfortunately did not efficiently discriminate the enantiomers [3]. As a second resource, capillary electrophoresis (CE) analysis was exploited for enantiomer resolution.

The CE is an electrodriven separation technique performed in a narrow-bore fused silica capillary filled with a background electrolyte (BGE). The mechanism of separation in CE is based on the different velocities of migration of charged species under the influence of a high-voltage electric field (100 to 700 V/cm) applied to the capillary. The migration velocity depends on the electrophoretic mobility of the species, which are related to their charge-to-size ratio. Although enantiomers have the same charge-to-size ratio, CE can be and is widely used for the separation of chiral molecules. This kind of CE separation is feasible by adding a chiral selector to the background electrolyte, a compound able to provide a differential interaction (enantioselectivity) between the enantiomers.

Here, the aim was the enantiomeric discrimination of 5-hexyl-2-methyl-3,4-dihydro-2*H*-pyrrole **2**, which was synthesized by the transamination/cyclization of 2,5-undecadione **1** applying commercial *S*-TA (ATA-251) or *R*-TA (ATA-117) from CODEXIS. It was simply accomplished by supplying a buffer solution, named the BGE, with a sulfobutyl ether-β-cyclodextrin as a chiral selector capable of discriminating the enantiomers of interest.

## 2. Results and Discussion

### 2.1. CE Analysis

The better binding between the (*S*)- or (*R*)-2 and sulfobutyl ether-β-cyclodextrin was evaluated using the CE technique. Among the available chiral selectors, the cyclodextrin (CD) derivatives are the most used additives for this purpose [4]. The separation is based upon a complex formation between the enantiomers and the chiral selector, resulting in the formation of labile diastereoisomers.

**Figure 2:**
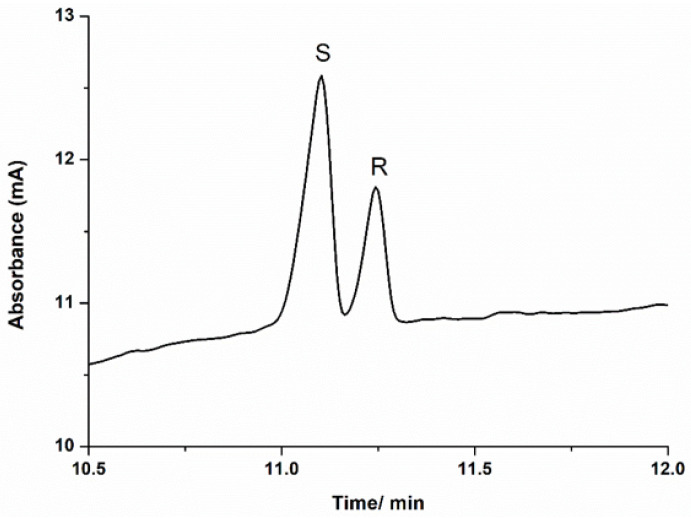
(**A**) Electropherogram of a mixture of (*S*)-(in excess) and (*R*)-5-hexyl-2-methyl-3,4-dihydro-2*H*-pyrrole. Separation conditions: bare fused silica capillary with a total length of 60 cm (51.5 cm effective) and 50 µm i.d.; background electrolyte of formate buffer (25 mmol L${}^{-1}$, pH adjusted to 3.8) and sulfobutyl ether-β-cyclodextrin sodium salt (3% m/v); hydrodynamic sample injection at 50 mbar for 5 s; separation voltage of −20 kV; UV detection at 210 nm.

Due to the different interaction of the enantiomers with the chiral selector, different time-dependent mobilities were obtained, so a capillary electrophoretic method for the enantioseparation of **2** was achieved, due to a favorable interaction between the sulfobutyl ether-β-cyclodextrin and the analytes. As shown in Figure 2, (*R*)- and (*S*)-pyrrolines could be base-line separated by CE using sulfobutyl ether-β-cyclodextrin as a chiral selector.

Although the pyrroline isomers were positively charged at the pH (3.8) of the BGE, these molecules migrated for the anode (positive end) because of their interaction with the cyclodextrins that are negatively charged. The peak with the highest intensity (peak area) was identified as the (*S*)-pyrroline because its concentration in the mixture was higher than the enantiomer of the (*R*) configuration (enantiomeric excess). The *S*-pyrroline showed a smaller migration time than (*R*)-pyrroline (Figure 2), indicating that the interaction between the isomer with (*S*) configuration and the sulfobutyl ether-β-cyclodextrin was more favorable. This result is consistent with the predicted model.

### 2.2. Docking Simulations

**Figure 3:**
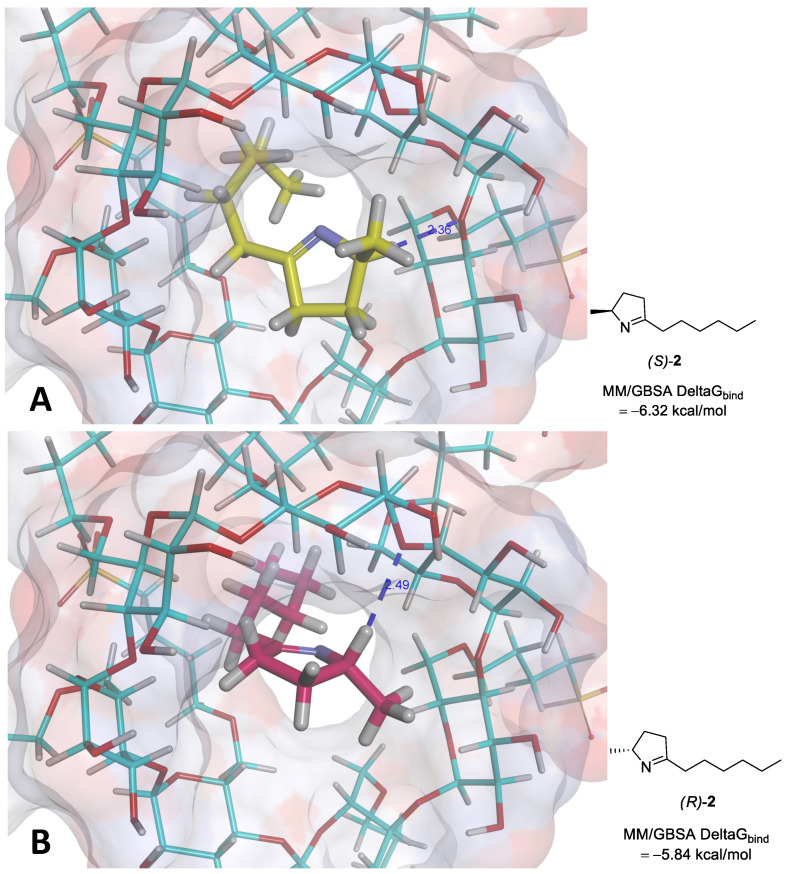
Proposed binding modes for the (*R*)- and (*S*)-enantiomers of 5-hexyl-2-methyl-3,4-dihydro-2*H*-pyrrole **2** with the sulfobutylether-β-cyclodextrin, highlighting the main intermolecular interactions and the corresponding binding free energies predicted using the Molecular Mechanics Generalized Born Surface Area (MM/GBSA) method: (**A**) (*S*)-pyrroline (carbon atoms in yellow; weak hydrogen bond of 2.36 Å); (**B**) (*R*)-pyrroline (carbon atoms in magenta; weak hydrogen bond of 2.49 Å).

Molecular docking simulations were performed to predict and rationalize the interactions (enantiomer recognition) between both enantiomers of **2** and sulfobutyl ether-β-cyclodextrin. The binding free energies ($\Delta G_{bind}$) for the docked poses of *S*-2 and *R*-2 with the sulfobutylether-β-cyclodextrin were estimated using the Molecular Mechanics Generalized Born Surface Area (MM/GBSA) method, a highly prevalent method for binding free energy prediction [5]. The estimated binding affinities suggest a slight preference of the sulfobutylether-β-cyclodextrin for the *S*-enantiomer, as the MM/GBSA $\Delta G_{bind}$ predicted for *S*-2 was −6.32 kcal/mol (Figure 3A), while the corresponding value observed for *R*-2 was −5.84 kcal/mol (Figure 3B).

Although these results suggest that the sulfobutyl ether-β-cyclodextrin would poorly discriminate the enantiomers, we must highlight that these values should not be considered as a direct replacement for in vitro results. Since the scoring functions of the docking programs were developed to be of general use, working across a large set of target proteins, this does not exclude the possibility that they may perform poorly for a particular drug target [6] or, in this case, a particular chiral selector. To confirm the feasibility of the predicted binding modes for the (*R*)-**2** and (*S*)-**2**, we also performed a visual inspection of the interactions between the best docking poses of each enantiomer and the chiral selector.

Analyses of the predicted interactions of the (*R*)-**2** and (*S*)-**2** with the sulfobutyl ether-β-cyclodextrin suggest a preferential interaction with the *S* enantiomer. As depicted in Figure 2, the only potential interactions performed for both enantiomers are nontraditional weak hydrogen bonds, where a carbon atom of the pyrroline acts as a hydrogen bond donor. The C - H ... A hydrogen bonds (where A = O or N) are considerably weaker interactions than the classical H-bonds, in spite of their recognized importance for the organization of the biological world [7].

In fact, they are believed to represent one-half of the strength of a traditional H-bond but are much more frequent in the process of ligand–protein interactions than generally believed [8]. In spite of both enantiomers displaying the same interaction, the shorter distance observed for the (*S*)-pyrroline (2.36 Å versus 2.49 Å) warrants a slightly stronger interaction, as highlighted by the binding affinities predicted in the molecular docking simulations.

Both enantiomers are predicted to interact with the target with similar binding modes, with their rings positioned at the entrance of the central cavity, while the alkyl chains enter the inner channel. Although the sulfobutyl groups do not seem to interact directly with the pyrrolines, they confer the geometry of a bulky cylinder to this cyclodextrin in contrast to the traditional β-cyclodextrin, where the side chains tend to form a floor in the structures, making their geometries resemble one half of a coconut.

Therefore, the ligands are predicted to be more loosely associated with the target, which reflects the lower predicted ΔG${}_{bind}$ values. However, it is important to notice that the manufacturer does not clearly state the actual substitution pattern or the configurations for the sulfobutyl ether-β-cyclodextrin [9], and we assumed for our simulations that all the hydroxyl groups of β-cyclodextrin would be substituted for the sulfobutyl ethers, a limitation that can seriously compromise the predictivity of this model in particular.

## 3. Experimental Section

### 3.1. General Methods

All commercially available reagents and solvents were purchased from Sigma-Aldrich or Alfa Aesar and used without further purification. TAs ATA-251 and ATA-117 were obtained from Codexis. Column chromatography was carried out using silica gel (Sigma-Aldrich, 230–400 mesh). Thin-layer chromatography (TLC) was performed on Merck Silica gel 60 F254 on aluminum foil and using a phosphomolybdic acid ethanolic solution as a spay staining reagent. ${}^{1}$H NMR (400.13 MHz) and ${}^{13}$C NMR (100.63 MHz) were acquired on a Bruker Avance III 400 (B0 = 9.4 T). Deuterated chloroform (CDCl${}_{3}$) or deuterated methanol (CD${}_{3}$OD) were used as a solvent, and their residual protic solvent was used as an internal reference. Chemical shifts are reported in δ (ppm), and the coupling constants (J) are reported in Hertz (Hz). GC analyses were performed on an Agilent 6850 GC (Agilent, Santa Clara, CA, USA) coupled with a flame ionization detector (FID), equipped with a fused silica capillary column HP-1 (30 m × 0.32 mm × 0.25 μm, Agilent) for general purposes or CP-Chirasil-DEX CB (25 m × 0.25 mm × 0.25 μm, Agilent) for enantiomeric discrimination. GC–MS analyses were performed on an Agilent 7890B chromatograph coupled with an Agilent 5977B mass spectrometer with an electron ionization source (EI) operating at 70 eV, and equipped with a fused silica capillary column HP-1ms (30 m × 0.25 mm × 0.25 μm).

### 3.2. Synthesis of (*R*)- and (*S*)-5-Hexyl-2-methyl-3,4-dihydro-2H-pyrrole **2**

Diketone 1 was synthesized based on a procedure previously described in [3]. A solution of 2-octanone (10.6 mmol) in THF (5.0 mL) was added dropwise to a solution of lithium diisopropylamine (2 M LDA in hexane, 6.35 mL, 12.7 mmol) in THF (40 mL) at −78 ${}^{\circ }$C. The resulting mixture was stirred for 30 min at −78 ${}^{\circ }$C. Chloroacetone (1.0 mL, 12.7 mmol) was added dropwise, and the reaction mixture was stirred for 20 min at −78 ${}^{\circ }$C and then allowed to warm to 0 ${}^{\circ }$C (30 min) and then to r.t. (2–4 h). The reaction was quenched by adding brine (15 mL) and extracted with dichloromethane (3 × 15 mL). The combined organic layers were dried over anhydrous MgSO${}_{4}$ and filtered, and the solvent was removed under reduced pressure. The crude product was purified by silica gel flash column chromatography (cyclohexane:ethyl acetate gradient 9.5:0.5 to 8:2) to afford diketone 2,5-decadione (1) in 74% yield (1.45 g). ${}^{1}$H NMR (400.13 MHz, CDCl${}_{3}$): δ 2.71–2.64 (m, 4H), 2.44 (t, *J* = 7.6, 2H), 2.18 (s, 3H), 1.61–1.53 (quint, *J* = 7.5, 2H), 1.35–1.20 (m, 6H), 0.88 (t, *J* = 7.3, 3H). ${}^{13}$C NMR (125 MHz, CDCl${}_{3}$): δ 209.7 (C), 207.3 (C), 42.8 (CH${}_{2}$), 36.9 (CH${}_{2}$), 36.0 (CH${}_{2}$), 31.4 (CH${}_{2}$), 30.0 (CH${}_{3}$), 28.8 (CH${}_{3}$), 23.5 (CH${}_{2}$), 22.5 (CH${}_{2}$), 13.9 (CH${}_{3}$). EI-MS (70 eV): 170 (M${}^{.+}$, 184), 127 (9), 114 (81), 99 (100), 71 (96), 55 (10).

The enzymatic reductive amination reactions were carried out in *Eppendorf* flasks containing the reaction mixture (450 μL of isopropylamine (1 mol/L) and pyridoxal phosphate (PLP, 1 mmol/L) in a triethanolamine buffer (100 mmol/L, pH 7.5 )) and 5 mg of *S*-TA (ATA-251) or *R*-TA (ATA-117). Then, 1,4-diketone **1** dissolved in DMSO (50 μL, 0.3 mmol/L) was added to the reaction mixture. The reactions were maintained at 30 °C and 200 rpm for 4 h and was finally interrupted by the addition of NaOH (50 μL, 5 mol/L), and extraction with ethyl ether (3 × 0.5 mL) afforded (*S*)-**2** or (*R*)-**2** in >99% yield and >99% *ee*. Subsequently, the organic fraction was analyzed by GC–MS.

### 3.3. Docking Simulations

The tridimensional structures of the enantiomers were built using the online version of Corina (https://www.mn-am.com/onlinedemos/corinademo) (accessed on 27 March 2021), which ascribes to 3-D structures pre-defined bond lengths and angles depending on the type of bond, the type of atom, and the hybridization state and defines the most probable torsional angles according to the nature of the structure [10,11]. The molecular geometries of the ligands were then refined via the standard energy minimization in Spartan 14 [12]. The structures of the sulfobutylether-β-cyclodextrin were drawn in Discovery Studio Visualizer 4.1 [13], by modifying the crystallographic model of the β-cyclodextrin (PDB ID: 1BFN). The geometries of the side chains of the resulting structure were then refined via energy minimization in Spartan 14 [12], while keeping the geometries of the D-glucopyranonsyl residues constrained. The lowest-energy conformer was then selected for the subsequent analyses.

For the molecular docking simulations, the protonation states for both ligands were assigned using the Epik module available in the Schrödinger suite 10.3. The binding free energies for the docked poses were then estimated using the Molecular Mechanics Generalized Born Surface Area (MM/GBSA) method available in the suite [14]. The most probable binding mode of each structure was then assigned based on the best pose for each enantiomer, the corresponding intermolecular interactions with the chiral selector being studied through visual inspection with the aid of the receptor–ligand interactions module of Discovery Studio Visualizer 4.1 [13].

### 3.4. Electrophoresis Procedure

The chiral CE separations were conducted using a 7100 Agilent CE system (Agilent Technologies, Waldbronn, Germany) equipped with a diode array detector (DAD). A bare fused silica capillary with a total length of 60 cm (51.5 cm effective) and 50 μm i.d. was used. The sample solutions were injected into the capillary by pressure (50 mbar for 5 s), and the separation voltage was −20 kV. During the separations, the temperature of the capillary cartridge was kept at 25 °C, and the UV detection was conducted at 210 nm. The BGE was composed of a 25 mmol L${}^{-1}$ formate buffer (pH 3.8, adjusted with NaOH solution) and 3% (m/v) sulfobutyl ether-β-cyclodextrin sodium salt. The capillary was conditioned daily by sequential flushes with methanol (10 min), a 0.1 mol L${}^{-1}$ HCl aqueous solution (10 min), ultra-pure water (2 min), a 1 mol L${}^{-1}$ H${}_{3}$PO${}_{4}$ aqueous solution (10 min), ultra-pure water (2 min), and finally with the BGE (10 min). The samples were diluted as required with the BGE before injection into the CE system.

## 4. Conclusions

In summary, the enantiomeric excess and absolute configuration in picomolar samples of (*R*) and (*S*)-5-hexyl-2-methyl-3,4-dihydro-2*H*-pyrroles were achieved by capillary electrophoresis with sulfobutylether-β-cyclodextrin (SB-β-CD) as a chiral selector.

## Data Availability

The data of this study are available in this article.
